# *Iridopsis socoromaensis* sp. n., a geometrid moth (Lepidoptera, Geometridae) from the Andes of northern Chile

**DOI:** 10.3897/BDJ.9.e61592

**Published:** 2021-01-28

**Authors:** Héctor A. Vargas

**Affiliations:** 1 Universidad Tarapacá, Arica, Chile Universidad Tarapacá Arica Chile

**Keywords:** Boarmiini, *Dalea pennellii*, Ennominae, Fabaceae, Folivorous larvae

## Abstract

**Background:**

*Iridopsis* Warren, 1894 (Lepidoptera: Geometridae: Ennominae: Boarmiini) is a New World moth genus mainly diversified in the Neotropical Region. It is represented in Chile by two described species, both from the Atacama Desert.

**New information:**

*Iridopsis socoromaensis* sp. n. (Lepidoptera: Geometridae: Ennominae: Boarmiini) is described and illustrated from the western slopes of the Andes of northern Chile. Its larvae were found feeding on leaves of the Chilean endemic shrub Dalea pennellii (J.F. Macbr.) J.F. Macbr. var. chilensis Barneby (Fabaceae). Morphological differences of *I. socoromaensis* sp. n. with the two species of the genus previously known from Chile are discussed. A DNA barcode fragment of *I. socoromaensis* sp. n. showed 93.7-94.3% similarity with the Nearctic *I. sanctissima* (Barnes & McDunnough, 1917). However, the morphology of the genitalia suggests that these two species are distantly related. The discovery of *I. socoromaensis* sp. n. highlights the need for additional surveys in underexplored areas to understand better the taxonomic diversity and evolutionary relationships of the mainly Neotropical moth genus *Iridopsis*.

## Introduction

The New World moth genus *Iridopsis* Warren, 1894 (Lepidoptera: Geometridae: Ennominae: Boarmiini) is mainly diversified in the Neotropical Region, from where 81 species have been described (Pitkin 2002, Vargas 2007), while 14 Nearctic representatives are known (Rindge 1966, Pitkin 2002). However, the taxonomic diversity remains insufficiently known in the Neotropical Region. For instance, 47 species were barcoded in a single small study area in southern Ecuador (Brehm et al. 2016). The genus is characterised morphologically by male genitalia with valva strongly divided into two lobes and the broad tegumen postero-laterally shaped like shoulders (Pitkin 2002). In a recent molecular phylogenetic study of the New World Geometridae, the species of *Iridopsis*, included in the analyses, clustered as a strongly-supported monophyletic group of the tribe Boarmiini sister to *Neofidonia* Warren, 1904 (*Perigramma* Guenée, [1858] + *Stenoporpia* McDunnough, 1920) (Brehm et al. 2019).

*Iridopsis* is represented in Chile by two described species, both from the Atacama Desert: *I. hausmanni* Vargas, 2007 from the transverse valleys near sea level and *I. parrai* Vargas, 2007 from the Pampa del Tamarugal, a plain at about 1000 m elevation between the Pacific coast and the western slopes of the Andes. The larvae of the two species feed on leaves of their respective host plants; *I. hausmanni* on *Haplorhus peruviana* Engl. and *Schinus molle* L. (Anacardiaceae) (Vargas 2014), *I. parrai* on *Prosopis tamarugo* Phil. (Fabaceae) (Vargas 2007). A third species, discovered in the highlands of the Andes of the country, is described and illustrated here.

## Materials and methods

The specimens examined were reared from folivorous larvae collected in March 2018 on Dalea pennellii (J.F. Macbr.) J.F. Macbr. var. chilensis Barneby (Fabaceae), near Socoroma Village at about 3300 m elevation on the arid western slopes of the Andes of northern Chile (18°16'41"S, 69°35'17"W). Genitalia dissections were performed using standard procedures. Images were captured with a Sony CyberShot DSC-HX200V digital camera attached to a Leica M125 stereomicroscope and a Micropublisher 3.3 RTV-QImaging digital camera attached to an Olympus BX51. The specimens studied are deposited in the “Colección Entomológica de la Universidad de Tarapacá” (IDEA), Arica, Chile.

One pupa was placed in ethanol (95%) and kept at -20°C to be used in genomic DNA extraction. This procedure was performed by staff of the “Laboratorio de Biología Molecular de Plantas” (Facultad de Ciencias Agronómicas, Universidad de Tarapacá, Arica, Chile) using the protocol described by Huanca-Mamani et al. (2015). DNA purification, PCR amplification and sequencing of the barcode fragment (Hebert et al. 2003) were performed in Macrogen Inc. (Seoul, South Korea) with the primers LEP-F1 and LEP-R1, following the PCR programme described by Hebert et al. (2004). Relationships of the obtained fragment were assessed using the BOLD Identification System (Ratnasingham and Hebert 2007).

## Taxon treatments

### Iridopsis socoromaensis

Vargas sp. n.

8E9AAAEC-AEEB-59F8-9864-3E3FC14B6A7E

A2BA204C-E2FE-4457-B1AB-1DFC470350FA

#### Materials

- **Type status:** Holotype. **Occurrence:** sex: female; otherCatalogNumbers: IDEA-LEPI-2020-025, genitalia slide HAV-1391; **Taxon:** order: Lepidoptera; family: Geometridae; genus: Iridopsis; specificEpithet: socoromaensis; taxonRank: species; scientificNameAuthorship: Vargas; nomenclaturalCode: ICZN; **Location:** continent: South America; country: Chile; stateProvince: Parinacota; locality: Socoroma; verbatimLocality: About 2 km south of Socoroma Village.; verbatimElevation: 3300; verbatimLatitude: 18°27’22’’ S; verbatimLongitude: 69°35’15’’ W; **Identification:** identifiedBy: Héctor A. Vargas; dateIdentified: 09 Nov 2020; **Event:** samplingProtocol: One female adult emerged in March 2019, reared from larva collected on Dalea pennellii var. chilensis in March 2018, pupa April 2018.; month: 2019; day: 3; verbatimEventDate: March 2019; **Record Level:** type: Physical Object; language: en; institutionCode: IDEA
- **Type status:** Paratype. **Occurrence:** sex: male; otherCatalogNumbers: IDEA-LEPI-2020-026, genitalia slide HAV-1236; **Taxon:** order: Lepidoptera; family: Geometridae; genus: Iridopsis; specificEpithet: socoromaensis; taxonRank: species; scientificNameAuthorship: Vargas; nomenclaturalCode: ICZN; **Location:** continent: South America; country: Chile; stateProvince: Parinacota; locality: Socoroma; verbatimLocality: About 2 km south of Socoroma Village.; verbatimElevation: 3300; verbatimLatitude: 18°27’22’’ S; verbatimLongitude: 69°35’15’’ W; **Identification:** identifiedBy: Héctor A. Vargas; dateIdentified: 09 Nov 2020; **Event:** samplingProtocol: One male adult emerged in March 2019, reared from larva collected on Dalea pennellii var. chilensis in March 2018, pupa April 2018.; month: 2019; day: 3; verbatimEventDate: March 2019; **Record Level:** type: Physical Object; language: en; institutionCode: IDEA
- **Type status:** Paratype. **Occurrence:** sex: male; otherCatalogNumbers: IDEA-LEPI-2020-027, genitalia slide HAV-1390; **Taxon:** order: Lepidoptera; family: Geometridae; genus: Iridopsis; specificEpithet: socoromaensis; taxonRank: species; scientificNameAuthorship: Vargas; nomenclaturalCode: ICZN; **Location:** continent: South America; country: Chile; stateProvince: Parinacota; locality: Socoroma; verbatimLocality: About 2 km south of Socoroma Village.; verbatimElevation: 3300; verbatimLatitude: 18°27’22’’ S; verbatimLongitude: 69°35’15’’ W; **Identification:** identifiedBy: Héctor A. Vargas; dateIdentified: 09 Nov 2020; **Event:** samplingProtocol: One male adult emerged in March 2019, reared from larva collected on Dalea pennellii var. chilensis in March 2018, pupa April 2018.; month: 2019; day: 3; verbatimEventDate: March 2019; **Record Level:** type: Physical Object; language: en; institutionCode: IDEA
- **Type status:** Paratype. **Occurrence:** sex: male; otherCatalogNumbers: IDEA-LEPI-2020-028, genitalia slide HAV-1392; **Taxon:** order: Lepidoptera; family: Geometridae; genus: Iridopsis; specificEpithet: socoromaensis; taxonRank: species; scientificNameAuthorship: Vargas; nomenclaturalCode: ICZN; **Location:** continent: South America; country: Chile; stateProvince: Parinacota; locality: Socoroma; verbatimLocality: About 2 km south of Socoroma Village.; verbatimElevation: 3300; verbatimLatitude: 18°27’22’’ S; verbatimLongitude: 69°35’15’’ W; **Identification:** identifiedBy: Héctor A. Vargas; dateIdentified: 09 Nov 2020; **Event:** samplingProtocol: One male adult emerged in March 2019, reared from larva collected on Dalea pennellii var. chilensis in March 2018, pupa April 2018.; month: 2019; day: 3; verbatimEventDate: March 2019; **Record Level:** type: Physical Object; language: en; institutionCode: IDEA

#### Description

**Figure 1.**
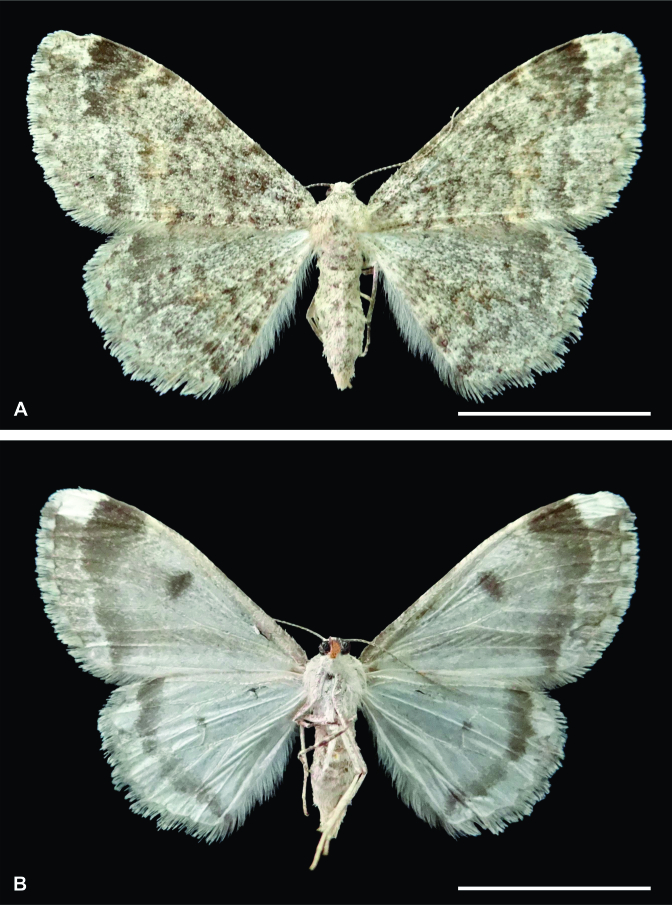
Female holotype of *Iridopsis socoromaensis* sp. n. **A.** Dorsal view; **B.** Ventral view. Scale bars 10 mm.

Female (Fig. 1). Forewing length 16.5 mm.

Head. Vertex and frons mainly creamy white with scattered dark grey scales. Antenna filiform with scape and pedicel creamy white, flagellum dark grey. Labial palpus dark grey with a few scattered creamy white scales.

Thorax. Mainly creamy white with scattered dark grey scales. Legs with creamy white and dark grey scales intermixed. Forewing dorsal surface with whitish-grey and dark grey scales intermixed; lines and discal dot slightly differentiated; postmedial line dark grey, triangular from costal margin to M3, slightly differentiated to inner margin; subterminal line narrow, creamy white, continuous with an apical concolorous blotch with a few scattered dark grey scales. Forewing ventral surface mainly whitish-grey from base to postmedial line; discal dot dark grey; postmedial line dark grey, narrowing from costal margin to inner margin; subterminal line narrow, creamy white, continuous with a concolorous blotch at apex; mainly dark grey between subterminal line and outer margin, lighter to tornus. Hindwing dorsal surface similar to forewing, but without a creamy white blotch at apex. Hindwing ventral surface mainly whitish-grey with a small dark grey discal dot and dark grey postmedial line.

Abdomen. Mainly creamy white with scattered dark grey scales.

**Figure 2.**
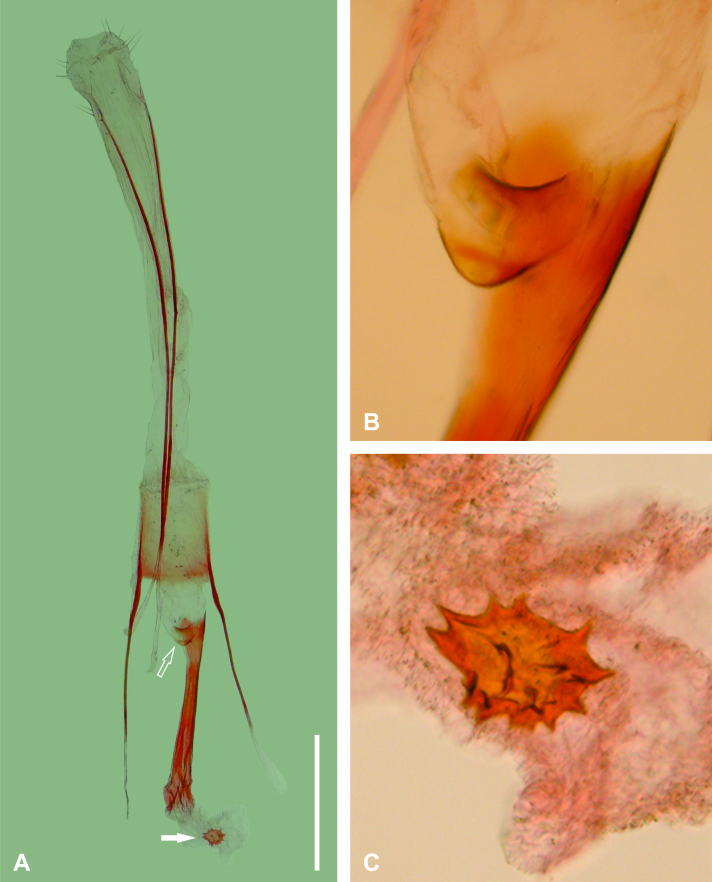
Female genitalia of *Iridopsis socoromaensis* sp. n. **A.** Ventral view; **B.** Detail of lamella antevaginalis, ostium and base of ductus bursae (open arrow of A); **C.** Detail of signum (solid arrow in A). Scale bar 1 mm.

Female genitalia (Fig. 2). Papillae anales lobe-like with a few setae. Posterior apophyses narrow, elongated, about seven times the length of papillae anales, apex reaching the basal part of the ductus bursae. Anterior apophyses narrow, slightly shorter than half the length of the posterior apophyses. Lamella antevaginalis a fine transversal stripe with anterior margin convex and posterior margin concave. Ductus bursae cylindrical, sclerotised, slightly shorter than anterior apophyses. Corpus bursae oval, membranous, length about a third that of the ductus bursae, signum sub-circular with serrated margin and a few additional small projections on the central area. Ductus seminalis on distal third of ductus bursae.

Male. Similar to female in size and maculation, but with bipectinate antenna and hair pencil on metatibia.

**Figure 3.**
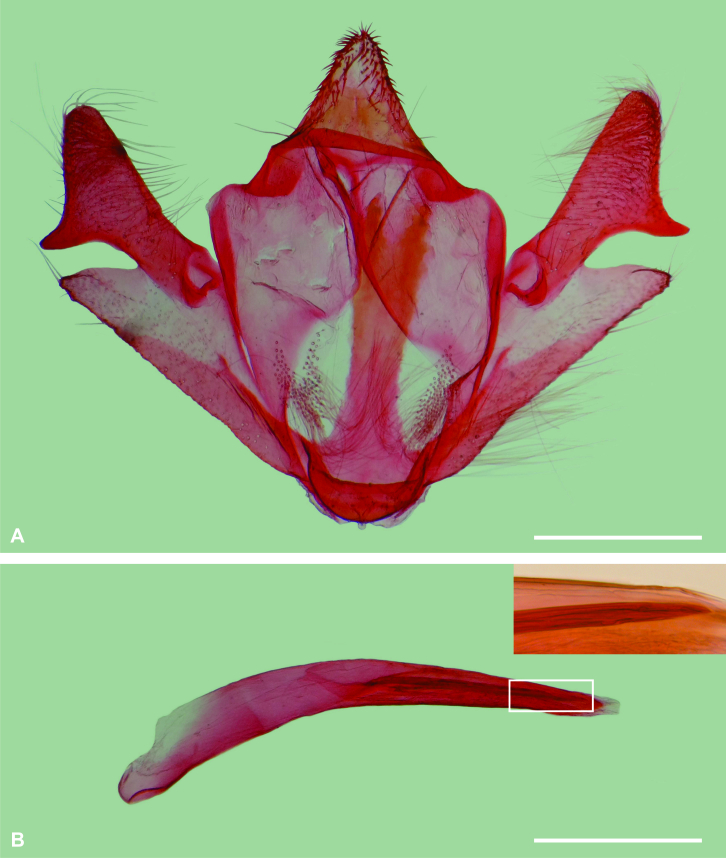
Male genitalia of *Iridopsis socoromaensis* sp. n. **A.** Ventral view, phallus removed; **B.** Phallus, lateral view; upper right rectangle shows apical portion of the cornutus (detail of rectangular area of B). Scale bars 0.5 mm.

Male genitalia (Fig. 3). Uncus triangular, round apex, with short setae near margin. Tegumen broad, anterior margin excavated in the middle. Saccus membranous ventrally. Juxta narrow, mainly as a longitudinal stripe; basal part semicircular slightly swollen antero-ventrally; apex excavated in the middle. Elongated hair-like scales near base of juxta. Valva deeply cleft; costal lobe wide, dorsal margin mainly straight, apex round, ventral process slightly curved outwards; sacculus triangular, apex round, internal face with a slightly differentiated longitudinal fold near apex. Phallus sub-cylindrical, about twice the length of the costal lobe of the valva, anterior part slightly downward-curved; a narrow, elongated cornutus on the vesica, slightly smaller than half the phallus length.

DNA barcoding. One sequence of DNA barcode (GenBank accession MW261921) of 658 bp was obtained. The BOLD Identification System found 93.7-94.3% similarity with the Nearctic *I. sanctissima* (Barnes & McDunnough, 1917), while similarity was 93.1% with the only haplotype of a Chilean congeneric (*I. hausmanni*) available in BOLD.

#### Diagnosis

*Iridopsis socoromaensis* sp. n. is recognisable by the dorsal surface of forewing with postmedial line like a dark grey triangle from the costal margin to M3 and an apical creamy white blotch with a few scattered dark grey scales, the female genitalia with lamella antevaginalis as a fine transverse stripe and the male genitalia with the costal lobe of the valva with a ventral process slightly curved outwards and the vesica with a single cornutus. In contrast, the two other Chilean representatives (*I. hausmanni* and *I parrai*) lack a dark grey triangle and apical creamy white spot on the forewing dorsal surface, lamella antevaginalis in the female genitalia and ventral process of the costal lobe of the valva in the male genitalia and the vesica has multiple cornuti. DNA barcode divergence with the only currently-available haplotype of *I. hausmanni* was found to be 93.1%. DNA barcode sequences of *I. parrai* remain unknown impeding comparisons.

#### Etymology

The specific epithet is derived from Socoroma Village, the type locality of *I. socoromaensis* sp. n.

#### Distribution

**Figure 4.**
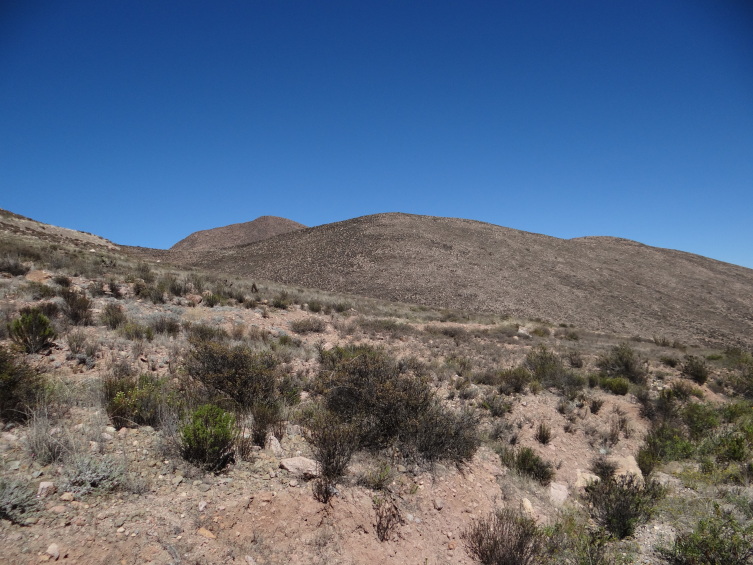
Habitat of *Iridopsis socoromaensis* sp. n. at the type locality near Socoroma Village, at about 3300 m elevation on the arid western slopes of the Andes of northern Chile (18°16'41"S, 69°35'17"W).

*Iridopsis socoromaensis* sp. n. is known only from the type locality near Socoroma Village in the Andes of northern Chile (Fig. 4).

#### Biology

**Figure 5.**
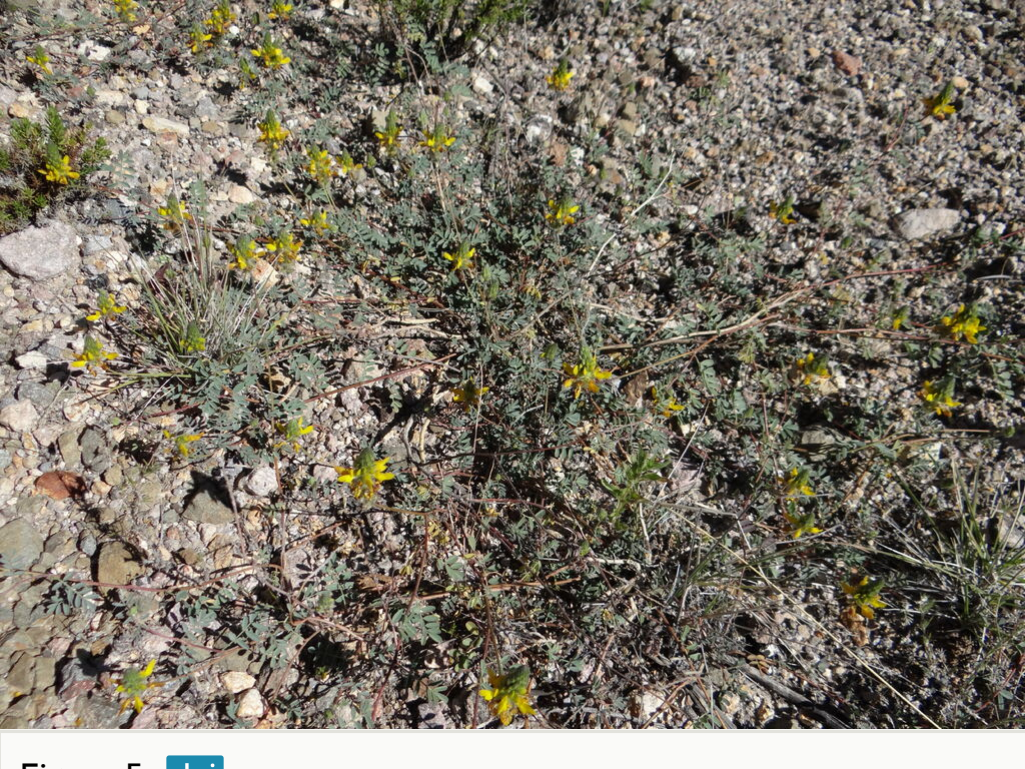
Host plant of *Iridopsis socoromaensis* sp. n., Dalea pennellii (J.F. Macbr.) J.F. Macbr. var. chilensis Barneby (Fabaceae), at the type locality.

The folivorous larvae of *I. socoromaensis* sp. n. were recorded on the Chilean endemic Dalea pennellii var. chilensis (Fig. 5), a fabacean prostrate shrub inhabiting a narrow elevation range between about 2500 and 3300 m and 18° and 20° S on the western slopes of the Andes (Rodriguez et al. 2018).

## Discussion

The Neotropical Region harbours a higher number of species of Geometridae than any other biogeographic region (Brehm et al. 2019). The wet tropical Andes are recognised as the main global diversity hotspot of this family, based on studies undertaken in southern Ecuador (Brehm et al. 2005, Brehm et al. 2016). In contrast, few species of Geometridae are known from the arid western slopes of the Andes of southern Peru and northern Chile. However, the recent discovery of *I. socoromaensis* sp. n. and other species (Cerdeña et al. 2019, Palacios et al. 2020, Vargas et al. 2020) suggests that native plants of these high elevation environments support an insufficiently-known fauna of geometrid moths.

*Iridopsis socoromaensis* sp. n. is the third species of the genus described from Chile. Although the phylogenetic relationships of the species of *Iridopsis* remain unknown, the morphology of the genitalia suggests a distant relationship between *I. socoromaensis* sp. n. and the two other Chilean representatives of the genus: *I. hausmanni* and *I. parrai*. In contrast, a close relationship between the latter two was suggested, based on some morphological characters of the male genitalia (Vargas 2007). Although DNA barcodes of *I. sanctissima* were the most similar to the sequence of *I. socoromaensis* sp. n., the similarity is lower than between closely-related species of Geometridae (e.g. Hausmann et al. 2009, Hausmann and Huemer 2011). Furthermore, the morphology also rules out a close relationship between them, because *I. sanctissima* has a distal cleft on the uncus, lacks a ventral process on the costal lobe, has a curved sacculus and vesica with several small cornuti, suggesting, instead, a close relationship with *I. clivinaria* (Guenée, 1857), another Nearctic congeneric (Rindge 1966). Additional surveys for geometrid moths in the arid high elevation environments of the Andes and other little explored environments of the Neotropics would be needed to find congeneric species close to *I. socoromaensis* sp. n.

Host plants remain unknown for a great number of species of Neotropical Geometridae. The available data suggest that host ranges are variable amongst species (Brehm 2002, Brehm 2003, Bodner et al. 2010). Although the Neotropical Region harbours the highest diversity of *Iridopsis* (Pitkin 2002), host plants are documented only for a few species involving a wide range of plant families (Brehm 2002, Brehm 2003, Vargas 2007, Vargas 2014, Marconato et al. 2008, Bodner et al. 2010, Robinson et al. 2010). A relatively-narrow host range has been suggested for *I. hausmanni*, with its only two records restricted to Anacardiaceae trees (Vargas 2014), while a wide host range has been described for the polyphagous *I. herse* (Schaus, 1912), whose larvae feed on plants of at least 17 families (Brehm 2002). Fabaceae host plants like *I. socoromaensis* sp. n. have, so far, been recorded for five other species of Neotropical *Iridopsis* (Brehm 2002, Vargas 2007).

The four specimens of *I. socoromaensis* sp. n. studied here lasted eleven months as pupae, from April 2018 to March 2019, suggesting pupal dormancy. Duration of the pupal stage from a few weeks to 27 months was described for another geometrid moth (*Eupithecia tarapaca* Rindge, 1987) that inhabits the same area (Vargas 2016). A long pupal duration with adult emergence around March (end of summer and beginning of autumn) in *I. socoromaensis* sp. n. could ensure abundant leaves of the host plant for larvae of this species, as the highest vegetation cover occurs after the summer rains in the study site (Muñoz and Bonacic 2006). However, additional observations are needed to understand better the natural history of *I. socoromaensis* sp. n. in the arid high elevation environments of the Andes.

## Supplementary Material

XML Treatment for Iridopsis socoromaensis

## References

[B6402265] Bodner F., Brehm G., Homeier J., Strutzenberger P., Fiedler K. (2010). Caterpillars and host plant records for 59 species of Geometridae (Lepidoptera) from a montane rainforest in southern Ecuador. Journal of Insect Science.

[B6402293] Brehm G. (2002). Diversity of geometrid moths in a montane rainforest in Ecuador.

[B6402310] Brehm G. (2003). Host-plant records and illustrations of the larvae of 19 geometrid moth species from a montane rain forest in Ecuador (Lepidoptera: Geometridae). Nachrichten des Entomologischen Vereins Apollo.

[B6402319] Brehm G., Pitkin L. M., Hilt N., Fiedler K. (2005). Montane Andean rain forests are a global diversity hotspot of geometrid moths. Journal of Biogeography.

[B6402328] Brehm G., Hebert P. D. N., Colwell R. K., Adams M. O., Bodner F., Friedemann K., Möckel L., Fiedler K. (2016). Turning up the heat on a hotspot: DNA barcodes reveal 80% more species of geometrid moths along an Andean elevational gradient. PLOS One.

[B6402345] Brehm G., Murillo-Ramos L., Sihvonen P., Hausmann A., Schmidt B. C., Õunap E., Moser A., Mörtter R., Bolt D., Bodner F., Lindt A., Parra L. E., Wahlberg N. (2019). New World geometrid moths (Lepidoptera: Geometridae): Molecular phylogeny, biogeography, taxonomic updates and description of 11 new tribes. Arthropod Systematics & Phylogeny.

[B6402367] Cerdeña J., Lopez E., Parra L. E., Vargas H. A., Farfán J. (2019). First record of *Pero
rodriguezi* Vargas, 2007 (Geometridae) in Peru with description of the female. Journal of the Lepidopterists’ Society.

[B6402386] Hausmann A., Hebert P. D. N., Mitchell A., Rougerie R., Sommerer M., Ewards T., Young C. J. (2009). Revision of the Australian *Oenochroma
vinaria* Guenée, 1858 species-complex (Lepidoptera: Geometridae, Oenochrominae): DNA barcoding reveals cryptic diversity and assesses status of type specimen without dissection. Zootaxa.

[B6402377] Hausmann A., Huemer P. (2011). Taxonomic decision as a compromise: *Acasis
appensata* (Eversmann, 1832) in Central Italy—a case of conflicting evidence between DNA barcode and morphology (Lepidoptera: Geometridae). Zootaxa.

[B6402407] Hebert P. D. N., Cywinska A., Ball S. L., deWaard J. R. (2003). Biological identification through DNA barcode. Proceedings of the Royal Society B Biological Science.

[B6402416] Hebert P. D. N., Penton E. H., Burns J. M., Janzen D. H., Hallwachs W. (2004). Ten species in one: DNA barcoding reveals cryptic species in the Neotropical skipper butterfly *Astraptes
fulgerator*. Proceedings of the National Academy of Sciences USA.

[B6402426] Huanca-Mamani W., Rivera-Cabello D., Maita-Maita J. (2015). A simple, fast, and inexpensive CTAB-PVP-Silica based method for genomic DNA isolation from single, small insect larvae and pupae. Genetics and Molecular Research.

[B6402435] Marconato G., Dias M. M., Penteado-Dias M. A. (2008). Larvas de Geometridae (Lepidoptera) e seus parasitoides, associadas à *Erythroxylum
microphyllum* St.-Hilaire (Erythroxylaceae). Revista Brasileira de Entomologia.

[B6402444] Muñoz A. E., Bonacic C. (2006). Variación estacional de la flora y vegetación en la precordillera andina de la comuna de Putre (I Región de Tarapacá, Chile) durante el período 2002–2003. Gayana Botánica.

[B6402453] Palacios C., Farfán J., Cerdeña J., Lazo-Rivera A., Parra L. E., Vargas H. A. (2020). A new species and a new record of *Pero* Herrich-Schäffer, 1855 (Lepidoptera: Geometridae) in the Andes of southern Peru. Nota Lepidopterologica.

[B6402465] Pitkin L. M. (2002). Neotropical Ennominae moths: A review of the genera (Lepidoptera: Geometridae). Zoological Journal of the Linnean Society.

[B6402474] Ratnasingham S., Hebert P. D. N. (2007). BOLD: the barcode of life data system (www.barcodinglife.org). Molecular Ecology Notes.

[B6402483] Rindge F. H. (1966). A revision of the moth genus *Anacamptodes* (Lepidoptera, Geometridae). Bulletin of the American Museum of Natural History.

[B6402492] Robinson G. R., Ackery P. R., Kitching I. J., Beccaloni G. W., Hernández L. M. HOSTS – a database of the world’s lepidopteran hostplants. http://www.nhm.ac.uk/research-curation/research/projects/hostplants/index.html.

[B6402501] Rodriguez R., Marticorena C., Alarcón D., Baeza C., Cavieres L., Finot V. L., Fuentes N., Kiessling A., Mihoc M., Pauchard A., Ruiz R., Sanchez P., Marticorena A. (2018). Catálogo de las plantas vasculares de Chile. Cayana Botánica.

[B6402519] Vargas H. A. (2007). Dos nuevas especies de *Iridopsis* Warren (Lepidoptera, Geometridae) del norte de Chile. Revista Brasileira de Entomologia.

[B6402528] Vargas H. A. (2014). First host plant records for *Iridopsis
hausmanni* Vargas (Lepidoptera, Geometridae) in the coastal valleys of northern Chile. Revista Brasileira de Entomologia.

[B6402537] Vargas H. A. (2016). First notes on the life history of *Eupithecia
tarapaca* Rindge (Geometridae) on the western slopes of the Andes of northern Chile. Journal of the Lepidopterists’ Society.

[B6402546] Vargas H. A., Hausmann A., Parra L. E. (2020). A new species of *Macaria* Curtis (Lepidoptera: Geometridae: Ennominae) from the Andes of northern Chile. Revista Brasileira de Entomologia.

